# Psychometric properties of an Arabic translation of the short entrapment scale in a non-clinical sample of young adults

**DOI:** 10.1186/s41155-024-00286-2

**Published:** 2024-01-18

**Authors:** Alfred Chabbouh, Elie Charro, Georges-Alain Al Tekle, Michel Soufia, Souheil Hallit

**Affiliations:** 1 Faculty of Medical Sciences, Lebanese University, Hadat, Lebanon; 2 School of Medicine and Medical Sciences, Holy Spirit University of Kaslik, P.O. Box 446, Jounieh, Lebanon; 3 Applied Science Research Center, Applied Science Private University, Amman, Jordan

**Keywords:** Entrapment, Suicide, Lebanon, Psychometric properties, Arabic

## Abstract

**Background:**

Entrapment is the feeling of wanting to leave an unbearable situation but believing that there are no options to do so. An Arabic entrapment Scale will assist healthcare professionals in the region in the prevention of suicide as the tool is tailored to the specific sociocultural context, which would enhance entrapment detection.

**Objective:**

In the current study, we aim to evaluate the psychometric properties of a translated Arabic version of the Entrapment Scale Short Form (E-SF).

**Methods:**

Three hundred eighty-nine Lebanese citizens were enrolled in this cross-sectional study.

**Results:**

The mean age of participants was 23.03 years (SD = 2.93), 69.4% being women. To examine the factor structure of the entrapment scale, we used an exploratory-to-confirmatory factor analysis (EFA-to-CFA) strategy. EFA and CFA results indicated that the fit of the unidimensional model of the Arabic Entrapment Scale (A-ES) was generally acceptable. Composite reliability of scores was adequate in the total sample (ω = .87). All indices suggested that configural, metric, and scalar invariance was supported across genders. Entrapment was positively and significantly correlated with suicidal ideation, alcohol use disorder, psychological distress, and orthorexia nervosa, suggesting convergent and divergent validity.

**Conclusion:**

The A-ES was found to be a valid and reliable tool to assess the degree of entrapment in Lebanese young adults. The A-ES will assist healthcare professionals in the region in the prevention of suicide as the tool is tailored to the specific sociocultural context, which would enhance entrapment detection.

## Background

Suicide is a public health issue. Deaths by suicide are far more prevalent in youth (ages 15–29) and middle and low-income countries (World Health Organization, 2014). From 2008 to 2018, suicide rates in Lebanon were estimated between 1.87 and 2.4 per 100,000 people, an underestimate due to underreporting (Bizri et al., 2021). Differences in gender, age, location, and social conditions characterize suicide (World Health Organization, 2014). Moreover, different risk factors may be associated with suicidal behavior, highlighting multiple pathways for suicidal ideation (Turecki & Brent, 2016). The Integrated Motivational-Volitional model (IMV) is one theory that proposes suicide in a three-part model. The first is the pre-motivational phase to which the diathesis-stress model is central. The second is the motivational phase where defeat, humiliation, and entrapment drive suicidal ideation under the influence of moderators like thwarted belongingness and perceived burdensomeness. The final phase is volitional where ideation becomes behavior under the influence of moderators like impulsivity and access to means (O’Connor & Kirtley, 2018).

Entrapment is the feeling of wanting to leave an unbearable situation but believing that there are no options to do so. It is divided into internal entrapment (feeling trapped within oneself) and external entrapment (having no way out of a current situation) (Teismann & Brailovskaia, 2020). Entrapment, in the absence of protective factors (e.g., future goals, belongingness), leads the individual to think of suicide as the only escape route. According to the IMV model, entrapment is the most proximal factor for suicidal ideations (O’Connor & Kirtley, 2018). Correlative studies have shown that both internal and external entrapment have a positive association with suicidal ideation (Forkmann & Teismann, 2017). It is a reliable predictor of suicide ideation and suicide attempts (Höller et al., 2022; Teismann & Brailovskaia, 2020).

To measure the degree of entrapment, Gilbert and Allan developed a 16-item Entrapment Scale (Gilbert & Allan, 1998). However, it was found to be too lengthy for clinical use. To solve this problem, De Beurs et al. shortened it to the 4-item Entrapment Short Form Scale (E-SF) which correlates almost perfectly with the original scale. E-SF includes two items measuring internal entrapment (“I feel trapped inside myself” and “I feel I'm in a deep hole I can't get out of”) and two items measuring external entrapment (“I often have the feeling that I would just like to run away” and “I feel powerless to change things”). E-SF is practical for use in clinical settings and can help clinicians and researchers differentiate between internal and external entrapment when necessary (De Beurs et al., 2020). To our knowledge, there exist no translations of the E-SF per se, however, the original 16-item scale has been translated and validated in German, Spanish, and Chinese (J. L. Ordóñez-Carrasco et al., 2021; Trachsel et al., 2010; Xu et al., 2021).

There has been no research on entrapment in Lebanon nor its connection to suicide. There is limited public awareness about mental disorders as well as cultural taboos around seeking treatment (Chahine et al., 2020). An Arabic entrapment Scale will assist healthcare professionals in the region in the prevention of suicide as the tool is tailored to the specific sociocultural context which would enhance entrapment detection (J. Ordóñez-Carrasco et al., 2020). In clinical practice, it is crucial to regularly incorporate entrapment beliefs into the risk assessments and therapeutic interventions. Addressing entrapment beliefs in psychotherapeutic interventions is another potential intervention that can help individuals develop coping strategies, and ultimately lower their risk of suicide (O’Connor & Portzky, 2018). Finally, the cultural stigma around suicide can make it difficult for individuals to speak openly about their thoughts and feelings related to suicide which can impede seeking help (El Majzoub et al., 2018). An Arabic entrapment scale may make it easier to assess suicidal risk by circumventing stigma as a barrier.

Therefore, in the current study, we aim to evaluate the psychometric properties of a translated Arabic version of DeBeurs et al.’s Entrapment Scale Short Form (E-SF). We expect this scale, herein referred to as the Arabic Entrapment Scale (A-ES), to show a robust association with suicidal ideation. With no scales available, the A-ES may prove crucial in the assessment of entrapment and suicidality among Arabic-speaking individuals and groups within diverse national and cultural contexts.

## Methods

### Procedures

This study received ethical approval from the Psychiatric Hospital of the Cross Ethics Committee (approval code: HPC-034–2022). The study was performed in accordance with the ethical standards as laid down in the 1964 Declaration of Helsinki and its later amendments or comparable ethical standards. Submitting the form online was equivalent to obtaining written informed consent from each participant. We collected the data via a Google Form link, between November and December 2022. We advertised the study on social media and included an estimated duration. Inclusion criteria for participation were being a resident and citizen of Lebanon with an age between 18 and 29 years. Each participant provided informed consent digitally. They were asked to complete the instruments described below presented in a pre-randomized order to control for order effects. In addition to the newly translated 4-item entrapment scale, we presented participants with tools to assess for suicidal ideations, psychological distress, alcohol use, orthorexia nervosa, and eating disorders. We aimed to assess the reliability, convergent validity, and factor structure of the entrapment scale. The survey was anonymous and its completion was voluntary and without remuneration.

### Participants

A total of 389 Lebanese citizens residing in the country at the time were enrolled in this cross-sectional study. As suicide is the leading cause of non-accidental death in youth (World Health Organization, 2014), only young adults aged between 18 and 29 years old were included in the study. The mean age of participants was 23.03 years (SD = 2.93), 69.4% being women (Table 1).

**Table 1:** Sociodemographic characteristics of the participants

Variable	Total sample (*N* = 389)	First subsample (*n* = 202)	Second subsample *(n* = 187)
Gender			
Men	119 (30.6%)	64 (31.7%)	55 (29.4%)
Women	270 (69.4%)	138 (68.3%)	132 (70.6%)
Age (in years)	23.03 ± 2.93	22.93 ± 2.90	23.13 ± 2.96

### Measures

#### Entrapment

We asked participants to complete a novel 4-item instrument, the Arabic Entrapment Scale (A-ES) that is a translation of De Beurs et al.’s Entrapment Scale Short Form (E-SF) (De Beurs et al., 2020). Items were rated using a Likert scale ranging from 0 (Not at all like me) to 4 (Extremely like me). A bilingual translator and a bilingual clinical psychologist forward and back-translated the tool. Both were native Arabic speakers that are fluent in English. The translations were put forth to a committee formed by mental health professionals and members of the general population who merged the translations and verified the adequacy and cultural appropriateness of the final translation. The latter was back-translated by a different bilingual translator and clinical psychologist. The committee compared the translations and resolved minor inadequacies. A pilot study of 30 participants was done to make sure all questions were clear.

#### Suicidal ideation

The Columbia-suicide severity-rating scale (C-SSRS) is a 10-item tool developed to assess suicidal ideation and behavior (Posner et al., 2011). We used the 5-item subscale of the Arabic version of the C-SSRS in this study. A higher score indicates more suicidal ideation (Chahine et al., 2020; Zakhour et al., 2021).

#### Psychological distress

The Depression Anxiety Stress Scale 8-Items (DASS-8) is a three-dimensional instrument that we used in this study to assess for symptoms of anxiety, depression, and stress. The 8-item instrument has proven adequate validity in the Arab population. Items are rated from 0 (does not apply to me at all) to 3 (applies to me very much, or most of the time) with a higher score indicating a higher level of psychopathology (Ali et al., 2021, 2022).

#### Alcohol use

We used the Arabic version of Alcohol Use Disorders Identification Test (AUDIT) that involves 10 items scored from 0 to 4. Total score ranges from 0 to 40 with a score of 8 and above indicating an alcohol use disorder. AUDIT has shown robust psychometric properties in Lebanese populations (Azzi et al., 2023; Hallit et al., 2020).

#### Orthorexia

To assess for orthorexia in participants, the obsession with eating healthy and proper food, we used the validated Arabic version of the Dusseldorf Orthorexia Scale (DOS). This is a 10-item scale rated from 1 (does not apply to me) to 4 (applies to me) with a higher score indicating higher levels of orthorexic behaviors (Rogoza et al., 2021).

#### Eating attitudes

We used the Arabic Eating Attitude Test (EAT-7) to assess participants’ disordered attitudes towards food. The scale is scored from 0 (never/almost never/infrequently) to 3 (always). A higher score indicates higher disordered eating attitudes (Fekih-Romdhane et al., 2022).

#### Demographics

Participants were asked to provide their demographic details consisting of age and gender.

### Analytic strategy

#### Data treatment

The dataset had no missing responses. To examine the factor structure of the entrapment scale, we used an EFA-to-CFA strategy (Swami & Barron, 2019). To ensure adequate sample sizes for both EFA and CFA, we split the main sample using an SPSS computer-generated random technique; sample characteristics of the two split halves are reported in Table 1. There were no significant differences between the two subsamples in terms of mean age, *t*(387) = 0.674, *p* = 0.501 and gender, *χ*^2^(1) = 0.236, *p* = 0.627.

#### Exploratory factor analysis

EFA is essential in early instrument development to determine latent factors of a scale (Tavakol & Wetzel, 2020). To explore the factor structure of the entrapment scale, we computed a principal component analysis with the first split-half subsample using the FACTOR software (Fornell & Larcker, 1981; Lorenzo-Seva and ten Berge, 2006). All requirements related to item communality were verified (Worthington & Whittaker, 2006), as well as those related to average item correlations, and item-total correlations (Clark & Watson, 1995). The Kaiser–Meyer–Olkin (KMO) measure of sampling adequacy and Bartlett’s test of sphericity ensured the adequacy of our sample (J. Hair, 2009). The procedure for determining the number of factors to extract was parallel analysis (using the Pearson correlation matrix) (Timmerman & Lorenzo-Seva, 2011). Weighted Root Mean Square Residual (WRMR) was also calculated to assess the model fit (values < 1 have been recommended to represent good fit) (Yu & Muthen, 2002). We retained items with “fair” loadings and above (i.e., ≥ 0.33) and those with low inter-item correlations (suggestive of low item redundancy) (Tie et al., 2022).

#### Confirmatory factor analysis

CFA is used to confirm the factor structure resulting from the EFA. It aims to see how well the data fits the proposed model (Tavakol & Wetzel, 2020). We used data from the second split-half to conduct a CFA of the model obtained in the EFA, using the SPSS AMOS v.29 software. The recommended minimum sample size required for conducting a confirmatory factor analysis is 3 to 20 times the number of the scale’s items (Mundfrom et al., 2005). Therefore, we assumed a minimum sample of 80 participants needed to achieve adequate statistical power based on a ratio of 20 participants per one item of the scale, which was exceeded in this subsample. We obtained parameter estimates using the robust maximum likelihood method and fit indices. Additionally, evidence of convergent validity was assessed using the average variance extracted (AVE), with values ≥ 0.50 considered adequate (Malhotra, 2011).

#### Gender invariance

To examine the gender invariance of entrapment scores, we conducted multi-group CFA using the second split-half subsample (Chen, 2007). We assessed measurement invariance at the configural, metric, and scalar levels (Vandenberg & Lance, 2000). Evidence of invariance was shown if ΔCFI ≤ 0.010 and ΔRMSEA ≤ 0.015 or ΔSRMR ≤ 0.010 (Chen, 2007; Cheung & Rensvold, 2002). We aimed to test for gender differences on latent entrapment scores using an independent-sample *t*-test only if scalar or partial scalar invariance were established.

#### Further analyses

We used McDonald’s ω and its associated 95% CI to assess composite reliability in the two subsamples. Values greater than 0.70 reflect adequate composite reliability (Dunn et al., 2014). We used McDonald’s ω to measure composite reliability because of established problems with the use of Cronbach’s α (McNeish, 2018). To assess convergent and criterion-related validity, we examined bivariate correlations between entrapment scores and those on the additional measures included in the survey (suicidal ideation, alcohol use disorder, disordered eating attitudes, and orthorexia nervosa) using the total sample. All scores had normal distribution, as identified by skewness and kurtosis values varying between − 1 and + 1 (F. H. Hair Jr. et al. 2017); therefore, the Pearson correlation test was used. Values ≤ 0.10 were considered weak, ~ 0.30 were considered moderate, and ~ 0.50 were considered strong correlations (Cohen, 1992).

## Results

### Exploratory factor analysis

#### Common variance

For the first split-half subsample, Bartlett’s test of sphericity, *χ*^2^(6) = 389.2, *p* < 0.001, and KMO (0.78) indicated that the entrapment items had an adequate common variance for factor analysis. The results of the EFA revealed one factor, which explained 71.06% of the common variance (item-factor loadings ≥ 0.80). The WRMR value was also adequate (0.15; 95% CI 0.12– 0.18), indicating a good fit of the model. The factor loadings are reported in Table 2.

**Table 2:** Factor loadings derived from the exploratory factor analyses (EFA) in the first split-half subsample, and standardized estimates of factor loadings from the confirmatory factor analysis (CFA) in the second split-half subsample. The first 2 items are external entrapment items. Items 3 and 4 assess internal entrapment

Item	EFA	CFA
1. I often have the feeling that I would just like to run away	.80	.68
2. I feel powerless to change things	.85	.78
3. I feel trapped inside myself	.87	.80
4. I feel I’m in a deep hole I can’t get out of)	.86	.73

#### Factor structure congruence and composite reliability

McDonald’s ω was adequate in women (ω = 0.89), men (ω = 0.79), and the total subsample (ω = 0.87).

### Confirmatory factor analysis

CFA with the second split-half subsample indicated that the fit of the unidimensional model of the Arabic Entrapment Scale (A-ES) was generally acceptable: *χ*^2^/df = 5.168/2 = 2.58, RMSEA = 0.092 (90% CI 0.001, 0.194), SRMR = 0.023, CFI = 0.989, TLI = 0.966. The standardized estimates of factor loadings were all adequate (see Table 2). The convergent validity for this model was adequate, as AVE = 0.56.

#### Composite reliability

Composite reliability of scores was adequate in women (ω = 0.87), men (ω = 0.82), and the total sample (ω = 0.87).

### *Gender invarianc*e

Next, we tested for gender invariance based on the unidimensional model of entrapment scores in the second split-half subsample. As reported in Table 3, all indices suggested that configural, metric, and scalar invariance was supported across gender. No significant difference was found in terms of entrapment scores between women (*M* = 5.44, SD = 3.80) and men (*M* = 5.75, SD = 4.25) in the second subsample, *t*(185) = 0.473, *p* = 0.637, *d* = 0.074.

**Table 3:** Measurement invariance across gender in the second split-half subsample

Model	χ^2^	*Df*	CFI	RMSEA	SRMR	Model Comparison	Δχ^2^	ΔCFI	ΔRMSEA	ΔSRMR	Δ*df*	*p*
Configural	8.66	4	.984	.079	.020							
Metric	10.72	7	.987	.054	.033	Configural vs metric	2.06	.003	.025	.013	3	.560
Scalar	11.54	10	.995	.029	.033	Metric vs scalar	0.82	.008	.025	< .001	3	.844

*Note. CFI* Comparative fit index, *RMSEA* Steiger-Lind root mean square error of approximation, *SRMR* Standardized root mean square residual

### Convergent and criterion-related validity

Entrapment was positively and significantly correlated with suicidal ideation, alcohol use disorder, psychological distress, and orthorexia nervosa. Importantly, the association was most significant (*p* < 0.001) between entrapment and suicide ideation as well as psychological distress. Entrapment scores were not significantly associated with eating attitudes or age (Table 4).

**Table 4:** Bivariate correlations between entrapment score and other measures included in the study and age

	1	2	3	4	5	6	7
1. Entrapment	1						
2. Suicide ideation	.50***	1					
3. Alcohol use disorder	.14**	.27***	1				
4. Psychological distress	.71***	.45***	.18***	1			
5. Orthorexia nervosa	.12*	− .01	.17**	.12*	1		
6. Eating attitudes	.07	.01	.07	.03	.32***	1	
7. Age	.04	− .002	.05	-.02	.03	.05	1

*Note.* *p < .05, **p < .01, ***p < .001

## Discussion

In this study, we set to translate the Entrapment Scale Short Form (E-SF) (De Beurs et al., 2020) into an Arabic Entrapment Scale (A-ES) after which we examined its psychometric properties in a sample of young adults aged 18–29. We hypothesized that this scale would show a robust positive association with suicidal ideation.

Using CFA and EFA, A-ES scores showed uni-dimensionality of the model with adequate factor loadings and composite reliability adequate for both men and women. Measurement of invariance across genders shows that the scale can be used in men and women. As certain assumptions for the use of Cronbach’s alpha are often violated in research and practice, we opted to use McDonald’s omega for its better measure of internal consistency (Deng & Chan, 2017). Our A-ES proved as reliable (ω = 0.87) as the E-SF that it was translated from (α = 0.87) (De Beurs et al., 2020).

The original Entrapment Scale Short Form (E-SF) showed that both a one-factor and two-factor model fit where the first factor was responsible for 50% of the variance (De Beurs et al., 2020). In our study, a one-factor model that accounted for > 70% of the variance was a better fit. This was similar to studies conducted on translated versions of the longer 16-item entrapment scale where one factor explains about 60% of the variance in the German study (Trachsel et al., 2010), 63% in the Spanish study (J. L. Ordóñez-Carrasco et al., 2021), and 70% in the Chinese study (Xu et al., 2021). When the 16-item entrapment scale was first conceptualized, it was thought that the reasons for the development of entrapment hold important significance (Gilbert & Allan, 1998) for which some later studies found that the scale was two-dimensional, consisting of external and internal entrapment (Cramer et al., 2019; Forkmann et al., 2018). This particularly holds true in the USA and UK samples as cultural differences have been postulated to be one of the reasons for the discrepancy in factor structure cross-nationally (Cramer et al., 2019; O’Connor & Portzky, 2018). For example, the studies in Germany, Spain, and China found that a single-factor solution is superior, which is what was observed in our Lebanese sample. As previously said, the effect may be at least partially culturally mediated but differences in study design, population studies, and samples are equally reasonable explanations as other UK and USA-based studies indeed found uni-dimensionality as the optimal fit (Forkmann et al., 2018; Oakey-Frost et al., 2022).

A-ES scores showed an extremely significant positive correlation with suicide ideation scores. Our results are consistent with the majority of studies (Li et al., 2018; O’Connor & Portzky, 2018; Shelef et al., 2016; Teismann & Brailovskaia, 2020; Wang et al., 2023) which may reflect the universality of the concept of entrapment across languages and cultures (regardless of factor structure). They were also consistent with the IMV model premise (O’Connor & Kirtley, 2018). Suicide is a worldwide issue that is subject to cultural differences as well as a product of different motives. For example, suicide may be considered a “solution” in certain cultures whereas a “sin” in others. Political, religious, economic, and sociocultural factors have a substantial impact on the perception of suicide (Goldsmith et al., 2002). However, we suggest that entrapment could be a common process that is less subject to cultural variability. Therefore, assessment of entrapment may prove valuable in sociocultural contexts where acknowledging the presence of suicidal ideation is challenging for a patient, such as in Lebanon (Mahfoud et al., 2011). However, for such a generalization to be true, it undoubtedly requires further international research focused on entrapment. So far, entrapment is a still-growing research field that has received attention only in certain countries like Germany, the UK, China, Spain, and the USA (De Beurs et al., 2020; Höller et al., 2020; Moscardini et al., 2022; J. L. Ordóñez-Carrasco et al., 2021; Wang et al., 2023).

A strong association between entrapment and psychological distress was expected. Entrapment was also associated with alcohol use disorder and orthorexia nervosa. A meta-analysis by Siddawi et al. highlighted the importance of entrapment across four psychiatric and alluded to the possibility of entrapment being a transdiagnostic construct (Ap et al., 2015). Another systematic review found similar findings, with entrapment and suicide being correlated in a manner that could not be solely explained by comorbid depression (Taylor et al., 2011). Hence, entrapment is an often under-investigated entity that could prove a clinically useful target in psychiatric and psychotherapeutic practices.

Regarding risk assessment, entrapment has been shown to be a strong moderator of suicide in various settings, itself moderated by variables like positive mental health and self-acceptance (Li et al., 2018; O’Connor & Portzky, 2018; Shelef et al., 2016; Teismann & Brailovskaia, 2020; Wang et al., 2023). As this is the first study regarding entrapment in Lebanon, further research should focus on establishing moderators of entrapment. More generally, studies regarding IMV model components such as defeat, humiliation, entrapment, suicidal ideation, and behavior (O’Connor & Kirtley, 2018) may all prove beneficial to establish the IMV model as cross-culturally valid. Furthermore, as current tools for assessing suicidal ideation often involve items targeting suicide thoughts and behaviors directly, may not accurately predict suicide risk in certain populations, and may fall short in assessing those uncomfortable discussing suicide directly (Lopez-Morinigo et al., 2018; O’Rourke et al., 2022; Stoven et al., 2018), we hypothesize that tools investigating suicide moderators like defeat, entrapment, and impulsivity may prove valuable in predicting suicide risk, notably in certain sociocultural contexts. We believe future research on risk assessment should focus on suicide moderators and buffers.

Finally, in our study, entrapment scores did not vary by age as our sample consisted exclusively of young adults aged 18 to 29. As suicide in this age group is on the rise, suicide prevention efforts are imperative (Busby et al., 2020). Psychotherapy based on targeting the feelings of entrapment has shown benefits in crisis intervention (Tzur Bitan et al., 2019). Adapting these strategies to specific cultural contexts may prove lifesaving.

## Limitations

Despite the general agreement of our results with other studies, certain limitations in our study must be acknowledged. First, due to the nature of the sampling method, it is unlikely that we captured a sample representative of the entirety of the Lebanese young adults or the population in general. This limits the generalizability of our findings. It would be valuable for future research to assess the replicability of our results in different age groups. Second, the online self-report nature of our survey opens our research to the well-known limitations of online surveys (Andrade, 2020). Future efforts should build upon our results to conduct entrapment research in clinical settings or with well-defined populations. Finally, we did not collect data about certain characteristics that could put specific subgroups (people living with disability, sexual and gender minorities, people with lower socioeconomic status, etc.) at higher risk for mental disorders and suicide (Honey et al., 2011; Moagi et al., 2021; Reiss, 2013). Researching entrapment in these subgroups could provide insight into whether they experience entrapment differently due to the specific societal barriers they face.

## Conclusion

The 4-item Arabic Entrapment Scale (A-ES) was found to be a valid and reliable tool to assess the degree of entrapment in Lebanese young adults. Entrapment scores were highly and positively correlated with suicide and psychological distress. They were also significantly and positively correlated with alcohol use and orthorexia nervosa. Future studies should focus on exploring moderators for the relationship between entrapment and suicide in the Lebanese population.

## Data Availability

All data generated or analyzed during this study are not publicly available due to the restrictions of the ethics committee (data are owned by the Psychiatric Hospital of the Cross). The dataset supporting the conclusions is available upon request to the corresponding author.

## Abbreviations

E-SF: Entrapment Scale Short Form
EFA: Exploratory factor analysis
CFA: Confirmatory factor analysis
A-ES: Arabic Entrapment Scale
DASS-8: Depression Anxiety Stress Scale 8-Items
AUDIT: Arabic version of Alcohol Use Disorders Identification Test
DOS: Dusseldorf Orthorexia Scale
EAT-7: Eating Attitude Test
KMO: Kaiser-Meyer-Olkin
AVE: Average variance extracted
